# LLMs augmented hierarchical reinforcement learning with action primitives for long-horizon manipulation tasks

**DOI:** 10.1038/s41598-025-20653-y

**Published:** 2025-10-21

**Authors:** Ning Zhang, Yongjia Zhao, Minghao Yang, Shuling Dai

**Affiliations:** 1 China’s State Key Laboratory of Virtual Reality Technology and Systems, Beihang University, Beijing, 100191 China; 2 Jiangxi Research Institute, Beihang University, Nanchang, 330096 China; 3 School of Artificial Intelligence, University of Chinese Academy of Sciences, Beijing, 100049 China

**Keywords:** Large Language Models (LLMs), Action primitives, long horizon manipulation, Hierarchical reinforcementlearning, Aerospace engineering, Mechanical engineering

## Abstract

Deep reinforcement learning methods have shown promising results in learning specific tasks, but struggle to cope with the challenges of long horizon manipulation tasks. As task complexity increases, the large state space and sparse reward make it difficult to collect effective samples through random exploration. Hierarchical reinforcement learning decomposes complex tasks into subtasks, which can reduce the difficulty of skill learning, but still suffers from limitations such as inefficient training and poor transferability. Recently, large language models (LLMs) have demonstrated the ability to encode vast amounts of knowledge about the world and to excel in context-based learning and reasoning tasks. However, applying LLMs to real-world tasks remains challenging due to their lack of grounding in specific task contexts. In this paper, we leverage the planning capabilities of LLMs alongside reinforcement learning (RL) to facilitate learning from the environment. The proposed approach yields a hierarchical agent that combines LLMs with parameterized action primitives (LARAP) to address long-horizon manipulation tasks. Rather than relying solely on LLMs, the agent uses them to guide a high-level policy, improving sample efficiency during training. Experimental results show that LARAP significantly outperforms baseline methods across various simulated manipulation tasks. The source code is available at: https://github.com/ningzhang-buaa/LARAP-code.

## Introduction

Robotic agents are increasingly deployed in various industries, from hospitals to production lines and high-precision assembly tasks^1^. Deep reinforcement learning (DRL) enables agents to autonomously acquire complex skills through continuous interaction with the environment. It has shown impressive performance in sequential decision-making and continuous control tasks, including autonomous driving and robotic manipulation^2–4^.

Although deep reinforcement learning has shown great results for simple manipulation tasks, it remains challenging to train the robotic agent to learn long horizon manipulation tasks, owing to the exploration trouble and task constraints^5^. Previous research in deep reinforcement learning has addressed the challenge of exploration by developing diverse temporal abstraction frameworks to capitalize on the hierarchical character of manipulation tasks^6–9^. On the other hand, alternative approaches have achieved notable success in training RL agents for manipulation tasks^2,10,11^ through careful engineering, effectively circumventing the exploration burden. Notably, Levine et al.^2^ utilize densely shaped rewards, Kalashnikov et al.^11^ use a comprehensive robotic infrastructure, and Andrychowicz et al.^10^ employ simulation-based training with carefully crafted reward functions for real-world transferability^12^.

Nevertheless, although these methods exhibit better scalability than basic DRL approaches, they often suffer from poor data efficiency, challenging reward design, limited generalization, and a lack of interpretability^13^. Humans can plan and execute temporally extended actions to accomplish long-horizon tasks in dynamic environments, often without explicitly reasoning about each individual action. A primary objective in robotics is to enable robots to learn and adapt like humans through continuous interaction with their environments. Consider the task of opening a door: “The person grasps the handle, rotates it, and opens the door.” This cognitive process involves selecting appropriate immediate actions to achieve a high-level goal (e.g., “grasping the handle”), receiving feedback on executed actions (e.g., “success”), and adjusting subsequent actions accordingly (e.g., “rotating the door”)^14^.

A common solution to these problems is hierarchical reinforcement learning (HRL), which reduces the search space by decomposing policies into high-level decisions (i.e., what the robot needs to do) and low-level executions (i.e., how to perform them using action primitives). Recent approaches have focused on designing or learning such hierarchical frameworks, ranging from manually constructing and refining action hierarchies^15,16^, to segmenting agent trajectories into discrete skills^17–20^, and, more recently, leveraging large-scale offline datasets to acquire skill libraries^21,22^.

Hierarchical reinforcement learning (HRL) mitigates some of the aforementioned challenges. However, as the range of available options or skills increases, these challenges tend to resurface. Although effective in specific settings, many of these methods depend on predefined planning domains, require complex reward functions, or rely on large task-specific datasets, which limits their scalability. In this work, we enhance exploration at both the high-level and low-level policy layers within the hierarchical framework.

Pre-trained large language models (LLMs), such as GPT-3 and ChatGPT, are trained on extensive text corpora to generate sequences in response to input prompts and exhibit strong multitask generalization capabilities^23^. Recent studies have explored the use of LLMs to convert high-level natural language commands into executable steps for long-horizon robotic manipulation tasks^24–29^. Due to training on massive text corpora, LLMs are capable of encoding broad world knowledge. We hypothesize that this knowledge can be leveraged to streamline the training of hierarchical policies, thereby substantially improving sample efficiency. Specifically, we investigate how large pre-trained language models can inject commonsense priors into hierarchical agents^30^.

Nevertheless, a key limitation of these approaches is the lack of assurance regarding which manipulation tasks large language models (LLMs) can effectively reason about and plan for, due to their limited real-world exposure during training. As a result, the action sequences generated by LLMs may lack contextual awareness of the robot’s environment and capabilities. To achieve long-horizon goals, the agent must perceive its environment, select relevant robotic skills, and sequence them appropriately. In this work, we assume the agent has access to a set of low-level skills. Given a high-level task description and the current state, the LLM directs the agent by proposing the most probable sequence of actions. Instead of relying on random exploration, we leverage these suggestions to guide exploration more efficiently. Meanwhile, decades of robotics research have developed a diverse set of functional modules tailored to specific robot behaviors, such as grasping^31^ and motion planning^32,33^. These predefined functional modules, known as behavior primitives, exhibit strong robustness and reusability in manipulation tasks such as object handling with the end-effector and collision-free motion planning. Prior work^5,12,34^ has significantly improved exploration efficiency and demonstrated promising results in learning manipulation skills by parameterizing the DRL action space. However, these methods struggle with skills that involve complex logic and temporal dependencies.

This paper presents a hierarchical reinforcement learning (HRL) framework that addresses long-horizon manipulation tasks by integrating guidance from large language models (LLMs) with pre-defined behavior primitives. To address the exploration challenges inherent in deep reinforcement learning (DRL), our approach leverages a library of high-level behavior primitives (e.g., grasping or pushing) in combination with low-level motor actions, enabling autonomous learning of a hierarchical policy. Given a high-level task description and the current state, the LLM guides the agent by recommending the most probable action sequences. Instead of relying on random exploration, we use these suggestions to guide exploration more efficiently. The main contributions of this work are summarized as follows:

1. We introduce a hierarchical reinforcement learning framework designed to tackle long horizon manipulation tasks through the integration of LLMs guidance and pre-established behavior primitives.
2. We develop a method to leverage LLMs for guiding exploration through the extraction of commonsense priors.
3. Extensive empirical evaluations demonstrate that our method significantly outperforms existing approaches in both learning efficiency and skill execution performance.

## Related works

### LLMs in robotics

As the research scope of large language models continues to expand, researchers have been progressively releasing a series of works on robots or embodied large language models^35–39^. Existing large language models are being applied in the field of robotics in various ways. Some directly utilize transformer models for end-to-end training^40^, while others employ fine-tuning of large language models using robot skill datasets^41^. There are also models focusing on addressing high-level decision-making tasks in robotics^42^, as well as those dedicated to solving three-dimensional trajectory planning problems^42^. Furthermore, large language models are being utilized for low-level motion planning tasks in robotics^41^, resulting in a series of embodied large language models. To better leverage the capabilities and knowledge of vision-language models in robotics, researchers integrated Google’s state-of-the-art language model, PaLM, with the cutting-edge visual model, ViT-22B. They utilized text and other multimodal data (primarily from robot sensors such as images, robot states, scene environment information, etc.) as inputs instead of pure text, and generated robot motion commands represented in text form as outputs. This approach facilitated end-to-end training, resulting in the development of the multimodal large model PaLM-E (Embodied) for robotic tasks^42^. As described in the work of PaLM-E, large language models have the capability to decompose high-level tasks into several semantically logical subtasks. However, due to the lack of real-world experience in LLMs, they cannot assess the potential impact of their outputs on the environment nor determine the actual state information of the environment and the robot, or whether the robot possesses the capability to execute these subtasks. Therefore, the seemingly logically subtask instructions generated by these models may not necessarily be smoothly executable by the robot in a real-world scenario. Therefore, the design logic of SayCan^25^ is straightforward: it divides the decision-making process of how the robot should execute tasks into two parts. “Say” represents the large language model (LLM), which outputs feasible high-level motion commands, while “Can” represents what the robot can do in the current environment. These two aspects are combined through a value function, jointly determining which instruction to select for actual execution. RT-1^40^ is an end-to-end control model for robots developed by researchers from Robotics@Google and Everyday Robots in 2022. Unlike traditional large language models, RT-1 primarily utilizes a transformer architecture, with a total parameter count of only 35 million. The transformer component comprises just 19 million parameters. It is a multitask robot control model specifically trained for robot operations. After releasing RT-1, researchers found that it lacked generalization ability and struggled to complete unseen tasks. Relying solely on manual demonstrations to gather more data for further training the RT-1 model proved to be costly and inefficient. RT-2^41^ is proposed to utilize a vision-language model (VLM) trained on internet-scale data directly for end-to-end robot control, enhancing both the generalization and semantic reasoning capabilities of robot operations. RT-2 has demonstrated that fine-tuning existing LLMs or VLMs with robot skill datasets can rapidly leverage the extensive generalization capabilities of VLMs, significantly improving the success rate and generalization ability of robot task execution. Octo^43^ is an open-source generalist robot policy trained on 800k trajectories, supporting various robotic platforms and controllable via language commands or goal images. Built on a large-scale Transformer architecture, it can be efficiently fine-tuned to new sensory inputs and action spaces within hours on standard consumer GPUs. Experiments across nine robotic platforms demonstrate its effectiveness as a versatile policy initialization for generalist robot learning.

Additionally, the context learning and intelligent prompting strategies supported by large language models have also been utilized in designing language-guided hierarchical policy agents. Wenlong Huang et al.^24^ investigates leveraging large language models to translate high-level natural language tasks into actionable steps, improving executability in interactive environments through semantic grounding and demonstration-based adaptation. Similarly, Michael Ahn et al.^25^ propose a method that integrates large language models with pretrained robotic skills, enabling robots to execute high-level natural language instructions by grounding semantic knowledge in real-world actions. Yuqing Du et al.^44^ propose ELLM, a method that leverages large language models to guide reinforcement learning exploration by rewarding agents for achieving language-suggested goals, improving common-sense behavior and downstream task performance. Murtaza Dalal et al.^45^ propose Plan-Seq-Learn (PSL), a modular approach that bridges high-level language and low-level control via motion planning, enabling robots to solve long horizon tasks from scratch and outperforming existing methods across multiple benchmarks. Our work, inspired by^30^, is the first to propose leveraging the planning capabilities of LLMs in conjunction with the learning ability provided by reinforcement learning (RL) to construct a hierarchical agent for solving long horizon tasks. This work further extends this idea by combining LLMs and behavior primitives to tackle more complex long horizon robotic manipulation tasks, thereby reducing the exploration burden of reinforcement learning algorithms.

### HRL

Deep reinforcement learning demonstrates outstanding performance by leveraging reward feedback for learning and optimization. Typically, it considers the ultimate goal as the target for optimization, leading to policy improvement. In the context of long sequence tasks, sparse reward functions can impede the learning process, resulting in subpar performance within traditional deep reinforcement learning frameworks. Hierarchical reinforcement learning decomposes the objective into multiple subtasks^46–48^, allowing the agent to incrementally learn to accomplish the overarching task by acquiring hierarchical policies, often leading to exceptional performance. The primary advantage of hierarchical reinforcement learning lies in its accelerated learning capability, reduced susceptibility to the curse of dimensionality, and robustness in addressing challenges associated with large state-action spaces. With its multi-level temporal abstraction capabilities and enhanced generalization abilities^15^, hierarchical reinforcement learning can simplify problem complexity, thereby facilitating the resolution of previously daunting tasks such as long horizon manipulation. For instance, Xintong Yang et al.^49^ proposed a unified hierarchical reinforcement learning framework known as the Universal Option Framework (UOF). This framework enables simultaneous training of upper and lower-level policies, leading to improved learning efficiency. However, it necessitates the manual decomposition of complex tasks and the implementation of a target generation mechanism. Dandan Zhang et al.^50^ introduce a method called Explainable Hierarchical Imitation Learning (EHIL), aimed at addressing challenges faced by service robots during the process of pouring drinks. Traditional deep imitation learning techniques suffer from black-box effects and dependence on demonstration data in this domain. However, EHIL overcomes these issues by establishing an interpretable task execution logic graph, enabling the robot to learn high-level general knowledge and perform low-level actions across different pouring scenarios. This framework not only improves the robot’s success rate, adaptability, and operability but also tracks the reasons for failures in an interpretable manner. Zhimin Hou et al.^50^ introduce a hierarchical reinforcement learning method that holds great promise for complex robot assembly control tasks. Traditional HRL algorithms often require policy learning, with each training step requiring resampling, limiting their performance in terms of data efficiency. This paper proposes a data-efficient HRL method that reformulates the enhanced Markov decision process (MDP) through policy-agnostic learning, enabling learning of both high-level and low-level policies from the same samples.

While the hierarchical reinforcement learning methods mentioned above have demonstrated certain successes, many practical applications necessitate collaborative execution. Existing hierarchical reinforcement learning methods often perform sub-tasks sequentially, resulting in inefficient learning and underutilization of data at the upper level during training. The above hierarchical reinforcement learning, while showing excellent performance, also faces the following issues: 1) The special structure of hierarchical reinforcement learning often makes it difficult to use old samples for new training. Adopting off-policy training can lead to the upper-level agent failing to learn effective hierarchical policies. Therefore, the common approach is to use on-policy training methods, but these methods suffer from the general problem of low sample utilization rates. 2) Due to the nature of hierarchical structures, the upper-level policy needs to wait until the lower-level policy approaches convergence before it can learn a stable hierarchical policy. As a result, hierarchical reinforcement learning often has lower learning efficiency since it cannot be trained synchronously with the lower-level policy. 3) Hierarchical reinforcement learning faces challenges in long sequential decision-making problems due to insufficient exploration capability, limiting its advantages. Recent studies have leveraged pre-built action primitives to expedite exploration in hierarchical reinforcement learning. Instead of designing low-level behavioral primitives, it is more effective to manually design behavior primitives and employ parameterization to achieve targeted action outputs. For example, Yuke Zhu et al.^5^ introduces Manipulation Primitives Enhanced Reinforcement Learning (MAPLE), which enhances standard reinforcement learning algorithms with a predefined library of behavior primitives to address the exploration burden in complex tasks. MAPLE significantly outperforms baseline methods in simulated manipulation tasks and demonstrates the ability to transfer policies to new task variants and physical hardware. Hao Wang et al.^13^ introduces Task-Driven Action Primitives Reinforcement Learning (TRAPs), which enhances the efficiency and effectiveness of robot learning in long horizon operation skills through formal methods and parameterized action spaces. TRAPs utilize linear temporal logic to specify complex operation skills and combine a predefined library of action primitives to improve the robot’s exploration efficiency. Empirical research demonstrates that TRAPs outperform most existing methods in terms of learning efficiency and effectiveness. Hao Zhang et al. build upon previous work by introducing TALD^51^, a temporal logic-guided affordance learning framework that enhances robotic manipulation through affordance-based contact prediction and LTL representation, improving task understanding and category-level generalization. Additionally, this paper emphasizes the integration of prior human knowledge into hierarchical reinforcement learning as a suitable approach. Consequently, this research combines the extensive common-sense knowledge of LLMs with HRL to address intricate robot long horizon manipulation tasks, thereby alleviating the strain on reinforcement learning algorithms and exploration efforts.

## Methods

**Fig. 1:**
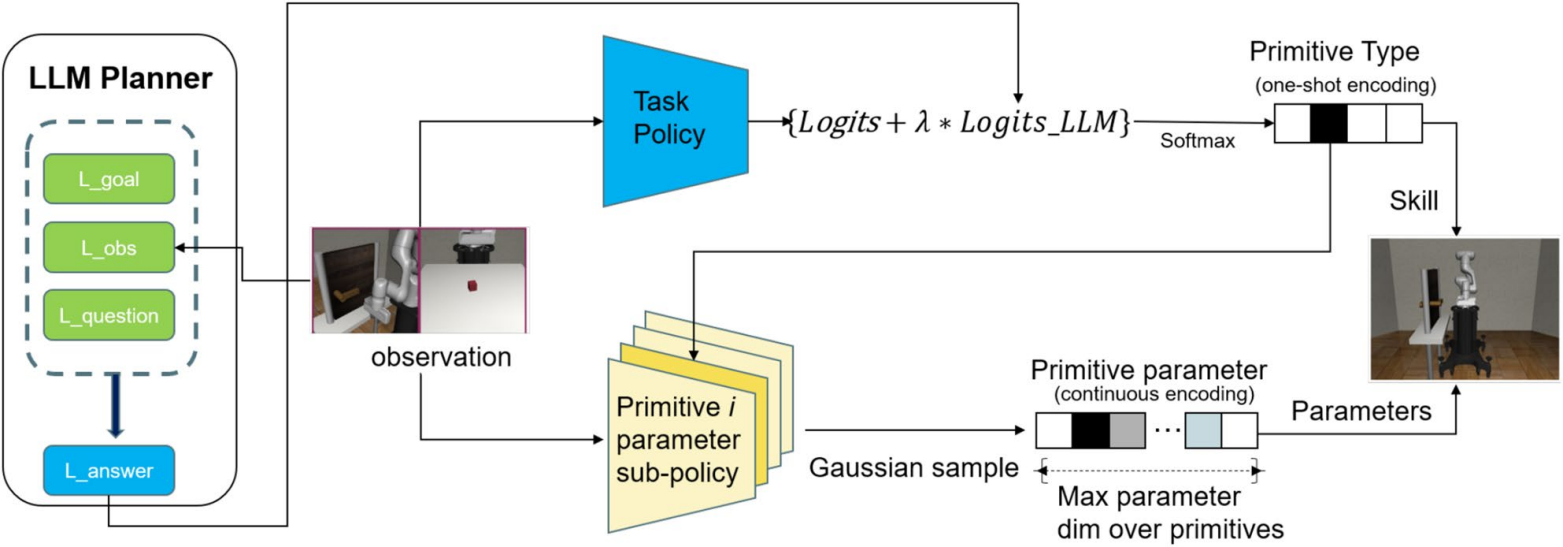
Framework of LARAP that enables the robot to leverage action primitives to solve manipulation tasks with LLMs guidance effectively and efficiently. The LLMs are used to guide the high-level policy and accelerates learning. It is prompted with the context, skill informance, and the current task and observation. The LLM’s output biases high-level action selection.

This paper introduces a new method called the LARAP, which effectively addresses long horizon operation tasks by integrating action primitives and LLMs technology. To overcome the difficulties of exploration and task learning in continuous action spaces, we break down the expected task into two components: “what” (predict subtask) and “how” (compute actions). The ‘what” aspect is managed by an RL task policy and LLMs guidance, while the “how” aspect is managed by a set of predefined action primitives. This setup allows the high-level task policy to consider the nature of the task by selecting primitives and their parameters while delegating detailed control to parameterized action primitives. Guided by the agent’s current state, we use an LLMs planner to enhance exploration within the high-level task policy. For more details, please refer to Fig. 1.

### Algorithm architecture

As depicted in Algorithm 1 and Fig. 1, the LARAP framework comprises three modules.

Env Module: A model dependent on the environment, responsible for preprocessing observations. In this study, the requisite environmental observations are directly acquired from integrated functions within Robosuite^52^.

LLMs Module: LLMs are utilized to augment task policy action selection through the utilization of common sense knowledge and planning capabilities. This occurs when the model is furnished with a task description and the current state is represented in natural language.

RL Module: An LLMs augmented hierarchical reinforcement learning framework responsible for determining action primitives and their corresponding parameters for execution within the environment. Essentially, the hierarchical reinforcement learning framework can be seamlessly integrated with deep reinforcement learning algorithm specifically designed for robot control tasks. In our study, the soft actor-critic (SAC) algorithm^53^, recognized as a state-of-the-art DRL approach, is chosen for its outstanding performance.

For implementation, LARAP receives environment observations as input and generates an action primitive along with its corresponding parameters to control the robot. The hierarchical framework described in^5^ is applied, wherein the RL module comprises both a high-level task policy and a low-level parameter policy. The task policy is depicted as a singular neural network, while the parameter policy is constituted by a series of subnetworks, with each subnetwork aligning with an action primitive. This organized framework enables us to accommodate primitives with diverse parameters. These primitive parameter sub-policy are crafted to facilitate batch tensor computation for action primitives with varying parameter dimensions. They all produce a uniform distribution for the parameters $x \in {R^{{d_A}}}$,where ${d_A} = \mathop {\max }\limits _a {d_a}$ represents the maximum parameter size across all action primitives. Throughout primitive implementation, the parameter *x* will be shortened to the size ${d_a}$ of the chosen primitive *a*. The visual depiction of LARAP can be seen in Fig. 1.

**Algorithm 1:**
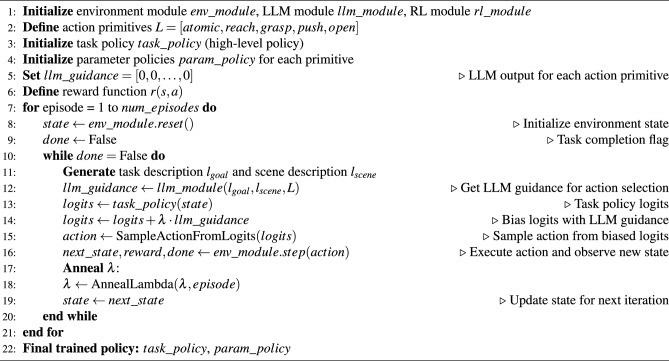
LARAP Framework for long horizon task execution.

We choose the SAC algorithm as the RL model in the hierarchical framework. We adapt the standard critic neural network ${Q_\theta }\left( {s,a} \right)$ and actor neural network ${\pi _\phi }\left( {a|s} \right)$ with our critic network ${Q_\theta }\left( {s,a,x} \right)$ and our hierarchical policy networks ${\pi _{ts{k_\phi }}}\left( {a|s} \right)$ and ${\pi _{{p_\psi }}}\left( {x|s,a} \right)$. The losses for the critic, task policy, and parameter policy are individually defined.

1 $$\begin{aligned} \begin{array}{l} {J_Q}\left( \theta \right) = ({Q_\theta }(s,a,x) - (r(s,a,x) + \gamma ({Q_{\bar{\theta }}}(s',a',x')\\ - {\alpha _{tsk}}\log ({\pi _{ts{k_\phi }}}(a'|s')) - {\alpha _p}\log ({\pi _{{p_\psi }}}(x'|s',a')))){)^2} \end{array} \end{aligned}$$

2 $$\begin{aligned} {J_{{\pi _{tsk}}}}\left( \phi \right) = \mathop E\limits _{a \sim {\pi _{ts{k_\phi }}}} \left[ {{\alpha _{tsk}}\log \left( {{\pi _{ts{k_\phi }}}\left( {a|s} \right) } \right) - \mathop E\limits _{x \sim {\pi _{{p_\psi }}}} {Q_\theta }\left( {s,a,x} \right) } \right] \end{aligned}$$

3 $$\begin{aligned} {J_{{\pi _p}}}\left( \psi \right) = \mathop E\limits _{a \sim {\pi _{ts{k_\phi }}}} \mathop E\limits _{x \sim {\pi _{{p_\psi }}}} \left[ {{\alpha _p}\log \left( {{\pi _{{p_\psi }}}\left( {x|s,a} \right) } \right) - {Q_\theta }\left( {s,a,x} \right) } \right] \end{aligned}$$

Here, ${\alpha _{tsk}}$ and ${\alpha _p}$ govern the maximum entropy objective for the task policy and parameter policy, correspondingly.

### Problem statement

We develop a system that interprets task instructions conveyed through natural language, akin to the approach discussed in^25^. The instructions may be lengthy, containing cautions and limitations, yet may not encompass all the required individual steps. We presuppose the agent possesses a limited number of skills and has authorization to access them, enabling sequential execution for handling long horizon manipulation tasks. With the limited options at hand, our goal is to formulate a high-level choice policy capable of making selections among these options.

Reinforcement learning learns from feedback rewards obtained through interaction between an agent and its environment, typically modeled using a Markov Decision Process (MDP). A Markov Decision Process is a five-tuple $M = (S,A,r,p,{p_0},\gamma )$ where *S* represents the set of all states in the environment, *A* represents the set of all actions in the environment, *r* represents the reward function, *p* represents the state transition function, and ${p_0}$ represents the initial state distribution. We expand this action space by incorporating a diverse collection of action primitives $L = \left\{ {{a^1},{a^2}, \cdots ,{a^k}} \right\}$ capable of executing behaviors that carry semantic significance.

Each action primitive $a \in L$ is formally depicted by a control module ${M_a}\left( x \right)$ that executes a limited, changeable series of atomic actions $\left( {{\mu _1},{\mu _2}, \cdots ,{\mu _t}} \right) ,{\mu _i} \in {R^{{d_{control}}}}$, where the specific action series are determined by input parameters $x \in {R^{{d_a}}}$. Here, ${d_a}$ represents the dimensionality of the input parameters to the action primitive *a*, which varies among various action primitives. To integrate these action primitives, we reframe our decision-making issue as a Parameterized Action MDP (PAMDP)^54^. In this framework, at each decision-making step, the robot performs a parameterized action $\left( {a,x} \right) \in A$ comprising the action primitive kind *a* and corresponding parameters *x*.

### Parameterized action primitives

The flexible parameterized action primitives act as the fundamental components for a wide range of robot manipulation tasks. In this study, we explore a primitive collection consisting of five primitives: 1) atomic; 2) reach; 3) grasp; 4) push; and 5) open. It’s noteworthy that incorporating input parameters with explicit semantics significantly enhances flexibility and utility when executing complex tasks. Yet, the predefined collection of action primitives may not be generally applicable across different environments. To tackle this issue, an extra atomic primitive is presented to bridge the discrepancy that cannot be addressed by other action primitives. The subsequent elaborates on the specifics of every motion primitive^5^.

1. Atomic: Executes a singular robot action.
2. Reach: Direct the end effector to a target position $\left( {x,y,z} \right)$ using the provided 3-D parameters. Execution may require up to 20 atomic actions.
3. Grasp: Position the end effector at a pregrasp location $\left( {x,y,z} \right)$ with a yaw angle $\theta$ determined by the provided 4-D parameters, followed by gripper closure. Execution may require up to 20 atomic actions.
4. Push: Position the end effector at an initial location $\left( {x,y,z} \right)$ with a yaw angle $\theta$, then displace it by $\left( {{\delta _x},{\delta _y},{\delta _z}} \right)$ based on the provided 7-D parameters. Execution may require up to 20 atomic actions.
5. Open: The end effector initiates a sequence of atomic actions to open its gripper autonomously, requiring no input parameters. Execution may involve up to 4 atomic actions.

We have applied these action primitives as hard-coded controllers, with each primitive requiring only a few lines of code. It’s important to note that these primitives accept input parameters of varying dimensions, operate over different temporal lengths, and yield distinct behaviors. These characteristics pose challenges for their integration within a learning framework. The following sections will discuss how to combine these primitives to solve long-sequence tasks.

### Using LLMs to guide high-level policies

This section presents our approach to leveraging LLMs to enhance exploration within the high-level policy framework of an HRL algorithm. LLMs enhance task policy action selection by leveraging common sense knowledge and planning abilities when provided with a task explanation and environment state represented in natural language. The fundamental concept involves utilizing LLMs to derive a value that approximates the likelihood of a particular action primitive being pertinent to accomplishing the overarching objective. As previously noted, each skill is paired with a textual description ${l_{skill}}$, and the current state of the environment is translated into natural language ${l_{scene}}$. Additionally, there exists a task description ${l_{goal}}$ detailing the objective the robot must accomplish, along with background information regarding the robot’s capabilities.

The LLMs are employed to assess the function ${f_{LLM}}\left( {{l_{skill}},{l_{goal}},{l_{scene}}} \right)$ of every skill during every task policy decision step. In essence, the LLM addresses the following inquiry: given the task ${l_{goal}}$ and the current scene description ${l_{scene}}$ , what is the likelihood of each skill being executed? The LLM output is a vector whose dimensions match the number of action primitives, and the sum of its elements equals 1. This specific analytical question-answering prompt has demonstrated superior effectiveness compared to open-ended prompts, as indicated by previous research^44^. After evaluating this process for each step, we obtain ${P_{LLM}} = \left[ {{p_1},{p_2}, \cdots ,{p_k}} \right]$. Dependence solely on ${P_{LLM}}$ is insufficient for resolving complex tasks. Simultaneously, employing RL and exploring devoid of any common-sense knowledge proves to be inefficient. Hence, while we continue to utilize RL and sparse rewards to acquire task policies, we also integrate common-sense priors, ${P_{LLM}}$, obtained from the LLMs prediction to help exploration. In the exploration policy, action selection involves sampling actions from a categorical distribution, with logits derived from the task policy head processing the state. The logits are biased using the LLMs common-sense prior ${P_{LLM}}$ and a weighting factor $\lambda$. Thus, the action selection process appears as follows: $a = Categorical\left[ {\pi \left( {{s_t}} \right) + \lambda \cdot {P_{LLM}}} \right]$. In this context, action *a* represents a predefined parameterized action primitive. The weight factor initiates at $\lambda = 1$ and gradually decreases through annealing until it reaches zero by the conclusion of the training process. This implies that the trained agent no longer relies on the LLMs throughout implementation.

**Fig. 2:**
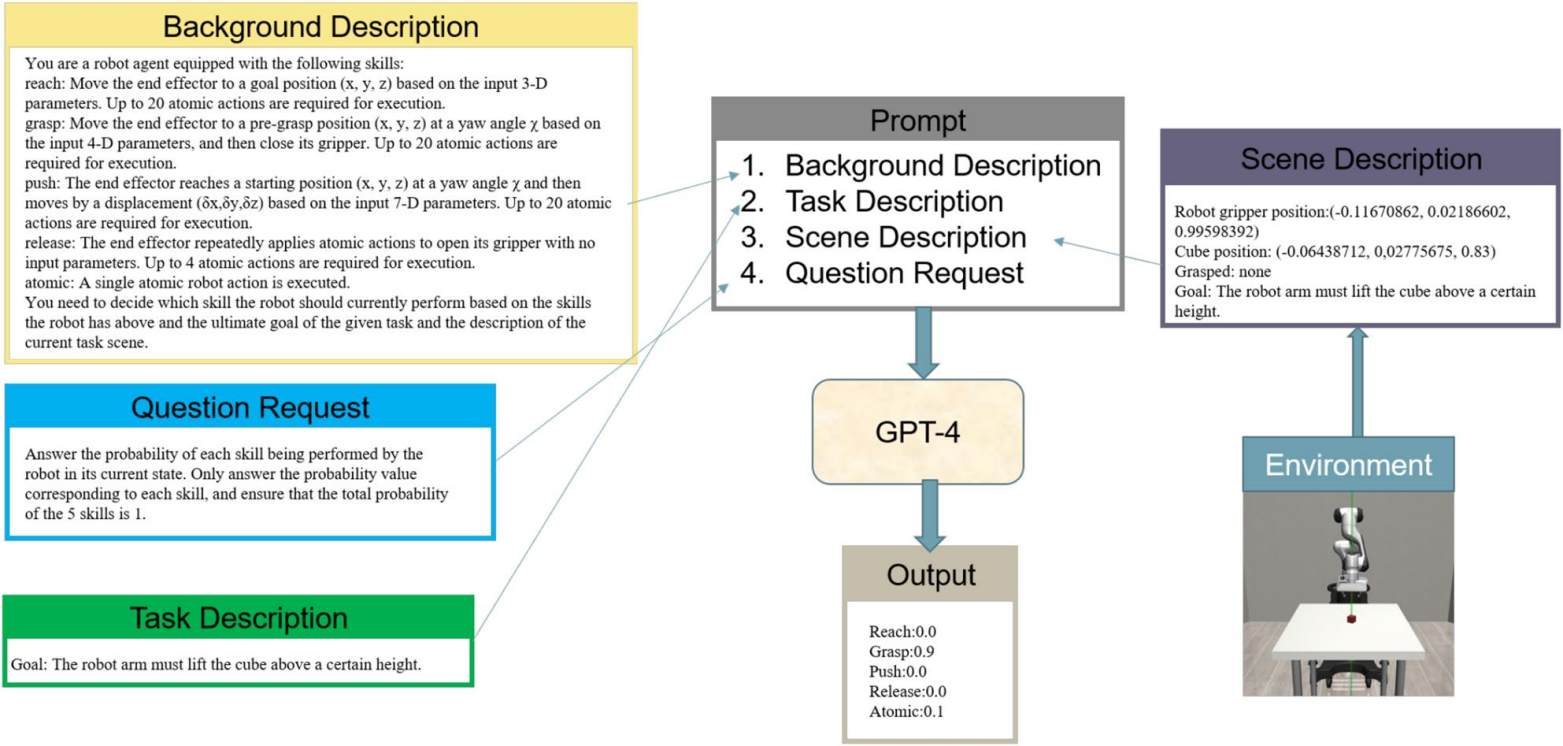
Detailed diagram of LLM guidance.

We employ GPT-4^55^ as our language model, renowned as one of the most capable LLMs accessible at the time of composing this document. We elaborate on the specifics of an example depicted in Fig. 2.

## Experiments

In this section, we assess LARAP in comparison to prior research. Extensive experiments are conducted, particularly focusing on: 1) Performance: examining whether LARAP surpasses prior approaches with regard to learning efficiency and effectiveness; 2) Expressiveness: assessing whether LARAP can enhance skill comprehension by leveraging LLMs, thereby offering superior guidance for the choice and mixture of action primitives to accomplish robot tasks; 3) Transferability: Examining whether LARAP can enhance learning efficiency when applied to meaningfully akin manipulation task.

### Experimental setup

Environments and Tasks: Robosuite^52^, a work designed for long horizon tasks emphasizing practical simulation and control, is utilized in this study to assess the efficiency of LARAP. For comparative analysis, six manipulation tasks of varying complexities outlined in robosuite are chosen. Detailed descriptions of the tasks are provided in Table 1. During every step, the agent will choose and execute an action primitive with particular parameters. It will then provide: 1) a reward feedback for agent learning; and 2) observations comprising the agent’s state and scene information in the environment. All evaluations are conducted on a desktop system running Ubuntu 18.04 equipped with an Intel Xeon(R) Gold 5120T CPU and an NVIDIA Quadro P5000 GPU.

**Table 1:** Robosuite task and task description.

Tasks	Task descriptions
Lift	Pick up a cube and lift it above the table.
Door Opening	Turn the door handle and open the door.
Pick and Place	Pick up a soda can and place it into a specific target compartment.
Stack	Stack a cube on top of another cube.
Nut Assembly	Fit a nut tool onto the round peg.
Cleanup	Push a jello box at the upper right corner and then store a spam can into a bin.

Baselines: The initial (and simplest) baseline is the standard SAC model^53^, which exclusively executes atomic primitives. Another notable baseline is manipulation primitive-augmented reinforcement learning (MAPLE)^5^, which constitutes an enhanced reinforcement learning framework built upon a collection of predefined action primitives. One approach to enhancing the efficiency of reinforcement learning and synthesizing task-conditioned policies involves generating task instructions using LLMs to guide high-level action selection. In this paper, we implement this approach to learn manipulation skills by extending MAPLE with the common sense knowledge and planning abilities of LLMs.

### Main experimental results

**Fig. 3:**
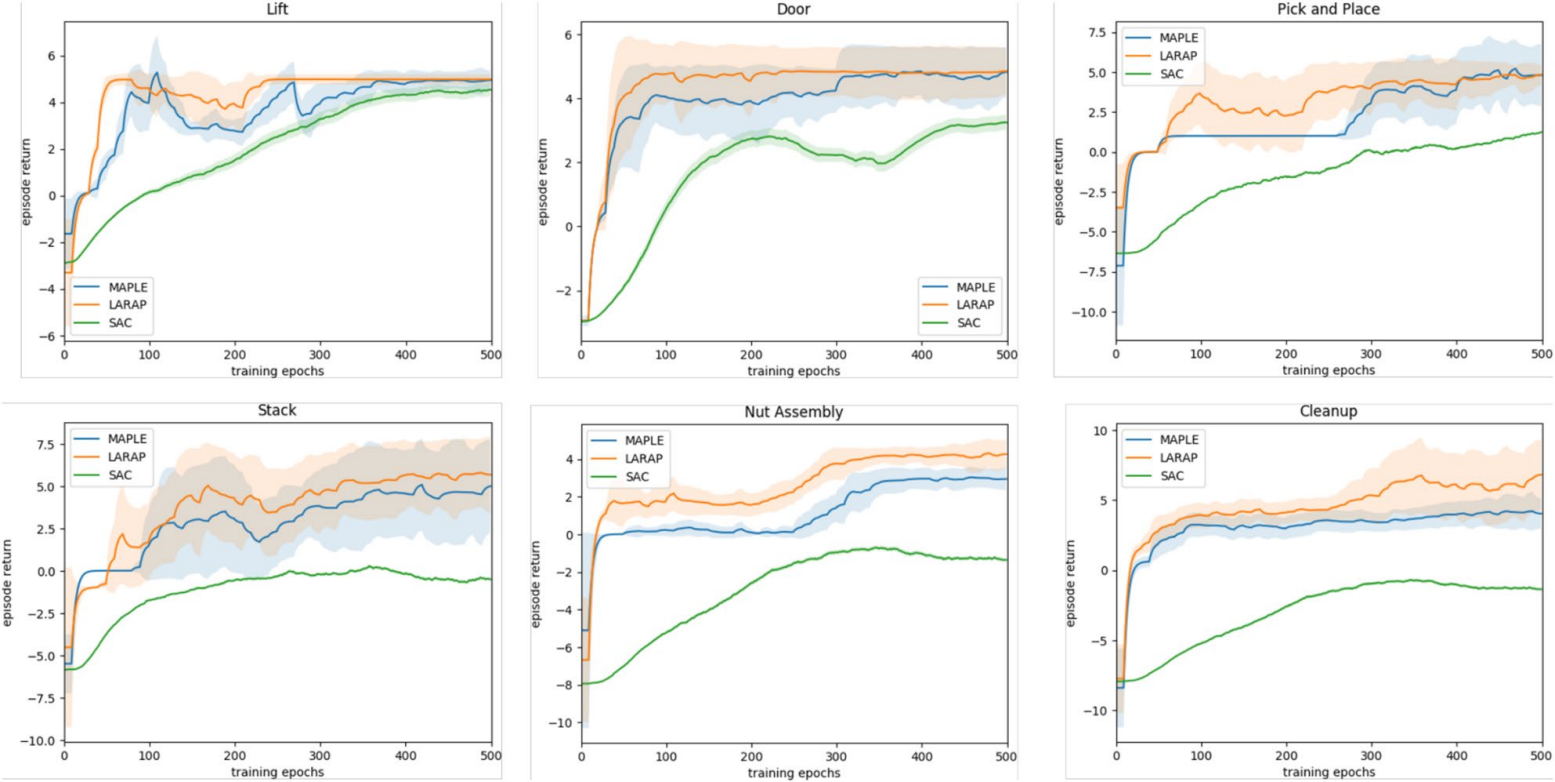
Learning curves depict the average episodic task rewards over the process of training. Results from all experiments are averaged across 5 seeds, with shaded regions indicating the standard deviation. The algorithm is evaluated every 10 epochs, and the curves are processed by smoothing functions.

In this section, LARAP is assessed regarding its performance, expressiveness, and transferability.

1. Performance: Both LARAP and the baseline approaches are utilized to execute the six manipulation tasks. Figure 3 illustrates the progression of rewards for the three primary baselines throughout the training process. The total training durations are mentioned in Table 2, with $\sim$ suggesting task learning breakdown. Observations reveal that 1) algorithms incorporating action primitives (MAPLE, LARAP) exhibit superior performance compared to those lacking action primitives (SAC), particularly evident when dealing with relatively complicated tasks (Door Opening, Pick and Place, Stack, Nut Assembly, and Cleanup). 2) LARAP can attain comparable performance to MAPLE for comparatively basic tasks (Lift, Door Opening, Pick and Place, and Stack), while demonstrating superior performance in complicated tasks (Nut Assembly and Cleanup). 3) LARAP exhibits greater learning efficiency than MAPLE, with this benefit becoming more pronounced as task complexity rises. This is attributed to our method of utilizing LLMs to enhance exploration in the high-level task policy based on the agent’s current state. 4) Furthermore, LARAP has the shortest training duration contrasted to other baselines, achieving over a 7.7$\%$ decrease compared to the algorithm without LLM (MAPLE).

**Table 2:** Total training time(hours).

Action Primitives	Lift	Door Opening	Pick and Place	Stack	Nut Assembly	Cleanup
SAC	101.35	104.20	$\sim$	$\sim$	$\sim$	$\sim$
MAPLE	107.37	114.30	140.32	120.54	152.40	125.26
LARAP	**100.48**	**103.59**	**129.74**	**111.38**	**140.29**	**116.24**

The achievement standards for task fulfillment outlined in^5^ are applied to further assess the efficacy of LARAP and all baseline algorithms. To evaluate the task execution success rate more comprehensively, we introduce three additional baseline algorithms: distributional soft actor-critic (DSAC)^56^, do as i can not as i say (SayCan)^25^, and waypoint-based reinforcement learning for robot manipulation tasks (WPRL)^57^. The compared methods can be categorized into three groups: traditional reinforcement learning approaches (SAC, DSAC), fully LLM-based approaches (SayCan), and hierarchical reinforcement learning approaches (WPRL, MAPLE, LARAP), where the low-level policies of MAPLE and LARAP are based on action primitives, while that of WPRL is based on intermediate waypoints. These baselines allow for a more thorough comparison, highlighting the effectiveness of the proposed LARAP method in long horizon manipulation tasks. The trained agent is assessed through 10 episodes, with the average of their success rates serving as our ultimate task success rate. The success rates are presented in Table 3. Initially, it is noted that LARAP attains the highest success rate among all baselines, reaching 100$\%$ in the majority of assessment tasks aside from Cleanup. While the success rate of MAPLE closely approaches ours, LARAP demonstrates a slightly superior overall success rate compared to MAPLE. As previously discussed, LARAP exhibits superior learning efficiency because of the guidance given by LLMs throughout the learning process. Secondly, methods incorporating action primitives (MAPLE, LARAP) demonstrate significantly better success rates compared to methods (SAC, DSAC) lacking action primitives. Particularly when dealing with complicated manipulation tasks (e.g., Nut Assembly and Cleanup), methods lacking action primitives struggle to achieve owing to the burden of exploration and task limitations. The experimental results demonstrate that the SayCan method is capable of accomplishing relatively simple manipulation tasks, such as door opening and pick-and-place. However, it struggles to handle more complex and long horizon tasks like Nut Assembly and Cleanup, often failing to generate coherent and executable action sequences. In contrast, WPRL, a reinforcement learning approach based on key waypoint planning, achieves performance comparable to our proposed LARAP method across both simple and complex scenarios. This indicates that incorporating task-relevant structural guidance, such as key path points, can significantly enhance task success rates in challenging robotic manipulation environments. In general, the results demonstrate that LARAP, which integrates RL with LLMs guidance and parameterized action primitives, can effectively learn a variety of manipulation tasks.

**Table 3:** Final skill success rate($\%$).

Action Primitives	Lift	Door Opening	Pick and Place	Stack	Nut Assembly	Cleanup
SAC	98.0$\pm$2.4	98.0$\pm$1.3	0.0$\pm$0.0	38.0$\pm$28.7	0.0$\pm$0.0	0.0$\pm$0.0
DSAC	99.0$\pm$3.6	98.0$\pm$2.8	14.0$\pm$4.7	43.0$\pm$5.4	0.0$\pm$0.0	0.0$\pm$0.0
SayCan	100.0$\pm$0.0	100.0$\pm$0.0	93.0$\pm$9.0	91.0$\pm$4.2	56.0$\pm$25.0	27.0$\pm$21.0
WPRL	100.0$\pm$0.0	100.0$\pm$0.0	100.0$\pm$0.0	99.0$\pm$1.5	98.0$\pm$2.3	93.0$\pm$2.1
MAPLE	100.0$\pm$0.0	100.0$\pm$0.0	95.0$\pm$7.7	98.0$\pm$2.4	99.0$\pm$2.0	91.0$\pm$5.8
LARAP	**100.0**$\pm$**0.0**	**100.0**$\pm$**0.0**	**100.0**$\pm$**0.0**	**100.0**$\pm$**0.0**	**100.0**$\pm$0.0	**94.0**$\pm$**1.2**

2) Expressiveness: Initially, we present the compositionality score from^5^, which serves as a measurable criterion for measuring the level of compositional behavior inside a trained policy. For a given task *T*, a collection of action sketches $\left\{ {{K^i}} \right\} _{i = 1}^n = \left\{ {a_1^i,a_2^i, \ldots a_{{T_i}}^i} \right\} _{i = 1}^n$ and available action primitives *L*, we calculate the compositionality of the agent’s behavior as the average pairwise normalized score between the task sketches.
  4 $$\begin{aligned} {f_{comp}}(T;L) = \frac{1}{{n(n - 1)}}\sum \limits _{i \ne j} {1 - \frac{{{d_{Lev}}\left( {{K_i},{K_j}} \right) }}{{\max \left( {\left| {{K_i}} \right| ,\left| {{K_j}} \right| } \right) }}} \end{aligned}$$
  where ${{d_{Lev}}\left( {{K_i},{K_j}} \right) }$ represents the Levenshtein distance^58^ between action sketches. In this study, it’s worth noting that every nonatomic primitive is considered a distinct token, and each instance of an atomic primitive is also treated as a distinct token. A better score indicates superior compositionality.

**Fig. 4:**
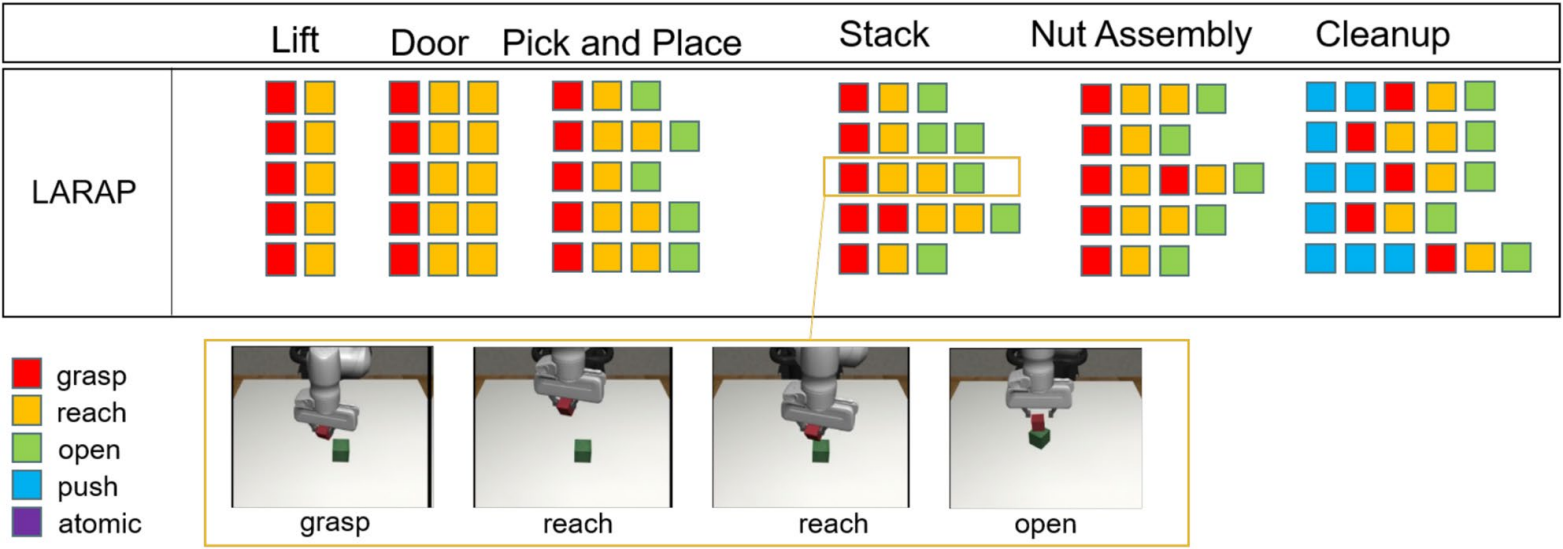
(Top) Visualization displays action sketches representing the learned policies of the agent employing LARAP across seven evaluation environments. Each row depicts a single sketch progressing sequentially from left to right over time. (Bottom) Visualization of action sketches and snapshots for stack.

The action sketches of LARAP with five different seeds are represented in Fig. 4. The compositionality scores are depicted in Table 4. Evidently, with a set of action primitives at its disposal, LARAP can effectively choose and combine suitable action primitives to accomplish a wide range of manipulation tasks. Furthermore, through the action sketch, people can easily notice the reason behind the choice of action primitives according to the task. Table 4 also demonstrates that LARAP achieves better compositionality scores compared to MAPLE, as the utilization of LLMs enables the robot’s choice of action primitives by guiding the high-level task policy.

**Fig. 5:**
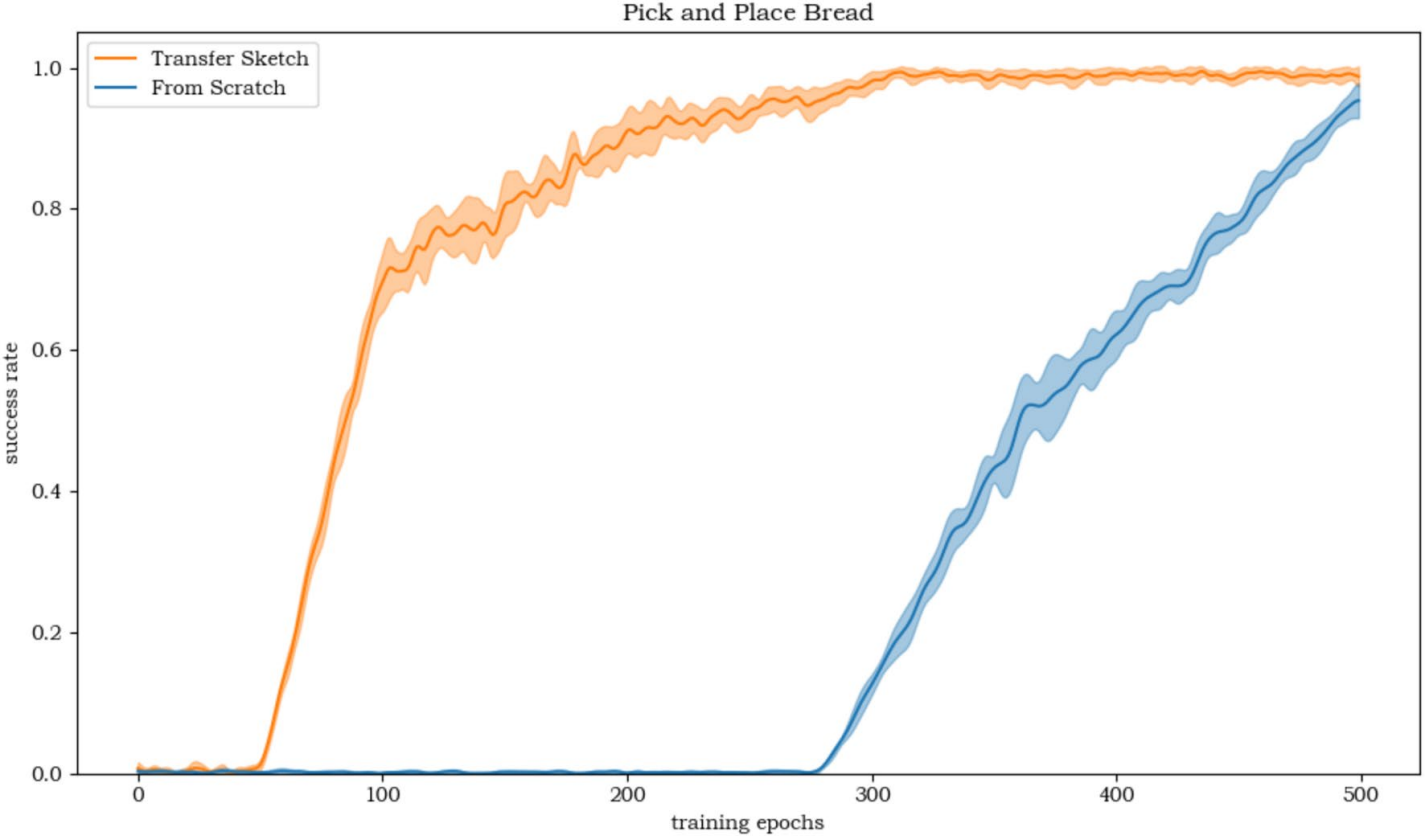
Ablation study on the impact of missing action primitives in the pick-and-place task.

**Fig. 6:**
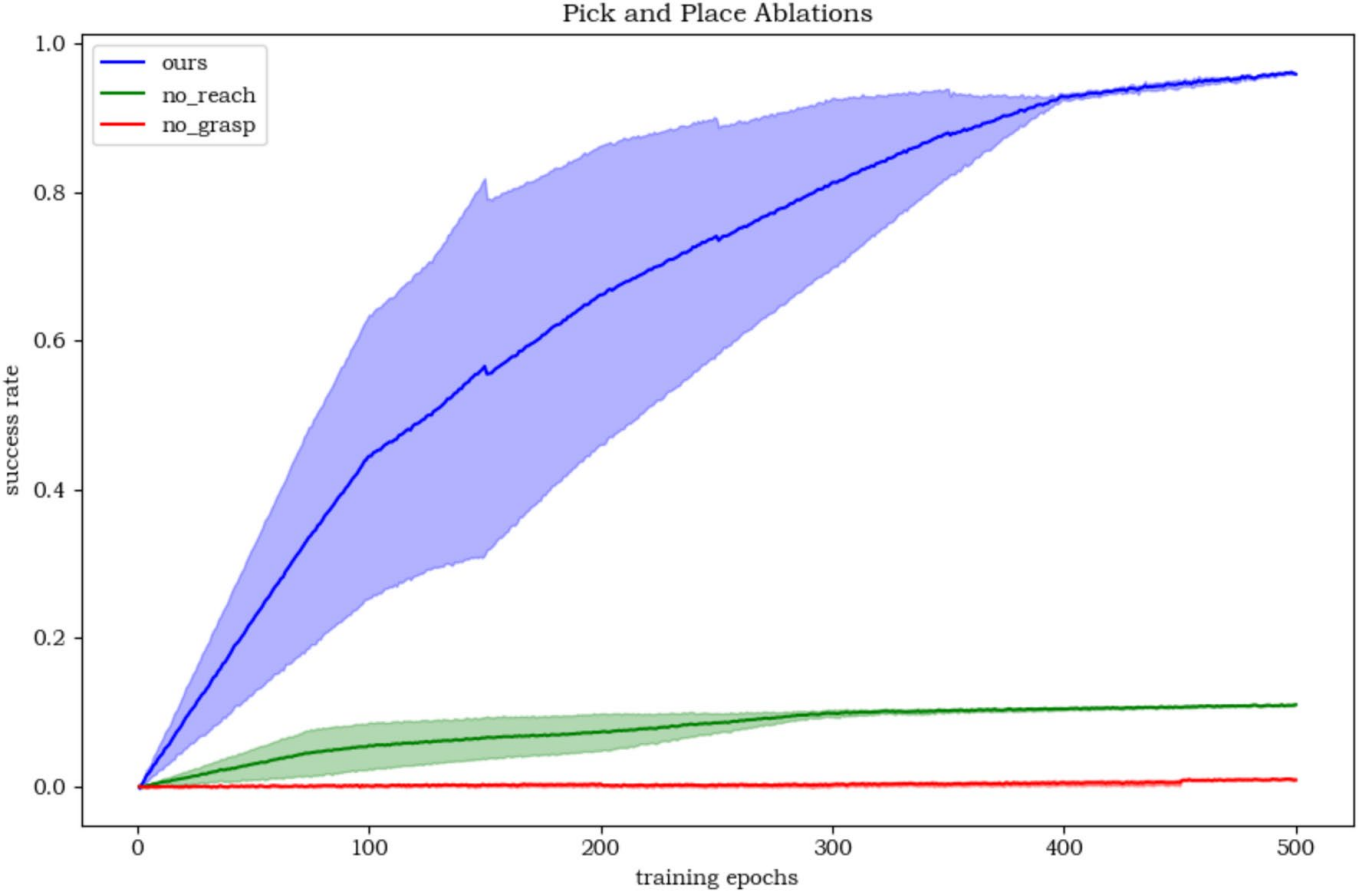
Success rate curves comparing learning from scratch and transfer-based policy learning in the Pick-and-Place Bread task.

3) Transferability: The expressiveness of LARAP forms the foundation for transferring policies to comparable tasks. When facing a new semantically comparable task, typically characterized by similar layouts of action sketches but differing parameters, updating the parameter policy suffices. This concept is exemplified through a Pick-and-Place scenario, wherein the task policy for picking and placing a soda can is transposed to a comparable task, albeit involving a distinct object (such as bread) and goal position. The achievement rate curvature for learning from scratch and transfer-based policy learning are illustrated in Fig. 6. Observations indicate that the transfer-based policy is more than two times as effective as learning from scratch. The experimental results demonstrate the potential for reusing trained action sketches or task policies in meaningfully akin tasks, enabling quick application to associated task variants.
4) Ablation Experiments: We conducted ablation experiments to evaluate the impact of the absence of individual action primitives on task learning. The objective of this experiment is to evaluate the impact on hierarchical policy learning when a specific action primitive is removed, thereby demonstrating the rationale behind the proposed combination of five action primitives. Specifically, we tested the pick-and-place task and compared the performance of our LARAP method with two ablated versions: 1) without the reach action primitive, and 2) without the grasp action primitive. As shown in Fig. 5, the removal of any single action primitive led to task failure. This highlights the crucial role of a proper combination of action primitives in our approach, which effectively alleviates the exploration burden and ensures successful task completion.

**Table 4:** Compositionality scores.

Action Primitives	Lift	Door Opening	Pick and Place	Stack	Nut Assembly	Cleanup
MAPLE	1.0	1.0	0.87	0.96	0.87	0.72
LARAP	1.0	1.0	**0.92**	**0.98**	**0.91**	**0.82**

## Conclusion

This study introduces LARAP for robot manipulation task learning, tailored specifically for mastering long horizon manipulation tasks. LARAP enhances the typical RL method in two key ways. Firstly, rather than relying solely on random exploration devoid of any prior knowledge, we utilize LLMs to recommend high-level task actions based on the task description and current state. Secondly, a preset collection comprising diverse action primitives, further enhances the effectiveness of agent exploration. Comprehensive practical research indicates that LARAP excels the majority of current algorithms. Furthermore, once the agent’s policy is successfully trained, the assistance of LLMs will no longer be necessary.

Building on the strengths of the proposed LARAP framework, several limitations and areas for future work remain. One notable limitation is that while LARAP significantly improves task learning efficiency, its performance heavily depends on the quality and accuracy of the high-level task descriptions and action primitives provided at the beginning. In cases where the task descriptions are ambiguous or incomplete, the framework’s performance may be compromised. Additionally, although the preset action primitive collection provides a solid foundation, it may not be sufficient to handle tasks that involve more complex or unforeseen actions. Future work could focus on integrating adaptive learning mechanisms that dynamically generate new action primitives based on task evolution, thus enabling the robot to handle more diverse and complex manipulation tasks.Another limitation is the lack of compliant force control in the current low-level policy, which directly outputs position commands. This may lead to excessive contact forces in contact-rich tasks, potentially causing failures or damage in real-world deployments. Incorporating compliant control strategies would be essential to ensure safe and robust execution.

## Data Availability

The data that support the findings of this study are available from “robosuite: A modular simulation framework and benchmark for robot learning” but restrictions apply to the availability of these data, which were used under license for the current study, and so are not publicly available. Data are however available from the first author upon reasonable request and with permission of “robosuite: A modular simulation framework and benchmark for robot learning”.
